# Mean Platelet Volume as a Predictor of Left Atrial Appendage Thrombus Resistance to Lysis in Patients With Atrial Fibrillation: Results of a 12-Month Follow-Up

**DOI:** 10.31083/RCM38943

**Published:** 2025-06-27

**Authors:** Tatiana P. Gizatulina, Alfira V. Belokurova, Aleksandra V. Mamarina, Natalia Yu. Khorkova, Elena A. Gorbatenko

**Affiliations:** ^1^Tyumen Cardiology Research Center, Tomsk National Research Medical Center of the Russian Academy of Sciences, 625026 Tyumen, Russian Federation

**Keywords:** atrial fibrillation, left atrial appendage thrombus, platelet, mean platelet volume, transesophageal echocardiography

## Abstract

**Background:**

Left atrial appendage thrombosis (LAAT), a contraindication to catheter ablation (CA), is a major problem in patients with non-valvular atrial fibrillation (AF). This study aimed to investigate the dynamics of LAAT and identify the factors associated with resistance to LAAT resolution over a 12-month period.

**Methods:**

A total of 83 of the 2766 patients with AF who underwent transesophageal echocardiography (TEE) before CA (median age, 62 years; 49 men) participated in follow-up studies. All patients received oral anticoagulants (OACs) and underwent a general clinical examination, which included a complete blood count, biochemical tests, and transthoracic echocardiography. In total, 39 patients (47%) had paroxysmal AF, and 44 patients (53%) had persistent AF.

**Results:**

Patients were divided into two groups based on dynamic TEE monitoring: Group 1 (n = 45), comprising patients whose LAAT resolved within 12 months, and Group 2 (n = 38), consisting of patients whose LAAT persisted until the end of the follow-up study. No significant differences were observed in age, sex, and incidence of cardiovascular disease between the groups. However, Group 2 patients were more likely to administer beta-blockers, diuretics, and rivaroxaban at the start of the study. The OACs were altered in 65 patients due to the repeated detection of LAAT. Comparative analysis revealed that Group 2 patients had higher right atrial volume index, N-terminal pro-brain natriuretic peptide (NT-proBNP) levels, mean platelet volume (MPV), platelet distribution width, and platelet–large cell ratio. Multivariate logistic regression analysis was used to derive a prediction model for LAAT resistance, which included three independent predictors: diuretics intake (odds ratio (OR) 3.800, 95% confidence interval (CI) 1.281–11.275; *p* = 0.016), NT-proBNP level (OR 1.001, 95% CI 1.000–1.001; *p* = 0.015), and MPV (OR 1.892, 95% CI 1.056–3.387; *p* = 0.032). The receiver operating characteristic (ROC) analysis confirmed the good quality of the model: Area under the ROC curve (AUC) 0.789, specificity 72.7%, and sensitivity 73.3%.

**Conclusions:**

This study confirmed that approximately 47% LAAT remains resistant to lysis 1 year after initial detection in patients with AF, regardless of the use of OACs. To our knowledge, this is the first time that platelet morphofunctional parameters, particularly MPV, have been identified as predictors of LAAT lysis resistance, and further research in this direction is needed.

## 1. Introduction

Atrial fibrillation (AF) is a prognostically unfavorable rhythm disorder accompanied by a fivefold increase in the risk of thromboembolic complications [1]. In non-valvular AF, thrombi most often form in the left atrial appendage (LAA), accounting for more than 90% of cases [2, 3]. With a sensitivity of 97% and specificity of 100%, transesophageal echocardiography (TEE) is considered the gold standard for the detection of left atrial (LA) thrombi [4]. According to studies, the cumulative prevalence of left atrial appendage thrombosis (LAAT) in patients with AF undergoing TEE and taking different oral anticoagulants is about 2.5–3.0% [5, 6]. Various guidelines recommend direct oral anticoagulants (DOACs) as a preferred anticoagulant option over vitamin K antagonists (VKAs) for stroke prevention in AF [1]; however, there is little data on optimal anticoagulant selection in patients with LAAT.

In their meta-analysis, Cheng *et al*. (2022) [7] showed that none of their inter-study variables significantly predicted the frequency of LAAT resolution for VKA and DOAC. Furthermore, the factors determining LAAT resolution or its persistence remain poorly understood. According to the results of another meta-analysis, the only variable that can be used to predict LAAT resolution is a higher rate of LAA emptying [6]. The studies included in this meta-analysis examined clinical, demographic, and echocardiographic characteristics as predictors of LAAT resolution in patients with non-valvular AF taking both VKAs and DOACs.

Given the important role of inflammation in the pathogenesis of AF, as well as the available data on the role of routine blood tests in the early diagnosis of cardiovascular diseases and their complications [8], we decided to study the features of hematological parameters as predictors affecting the resolution of LAAT. Hematological parameters obtained as part of a complete blood count (CBC) mainly include the classification and quantification of white blood cells (WBCs), red blood cells (RBCs), and platelets (PLTs). Based on previous studies in which routine hematological parameters were used as predictors of stroke or LAAT in patients with AF [9, 10], we hypothesized that in addition to clinical and echocardiographic characteristics, hematological parameters may serve as new predictors of LAAT resistance to lysis.

The aim of the study was to investigate the dynamics of LAAT and identify factors associated with resistance to LAAT resolution over a 12-month period.

## 2. Materials and Methods

This study was an open, single-center prospective study. The study adhered to the guidelines outlined in the revised 2013 Helsinki Declaration, and informed consent was obtained from all patients individually. The study was supported by the Ministry of Education and Science of the Russian Federation (Project No. 122020300112-4). The study protocol received approval from the Scientific Ethics Committee of the Tyumen Cardiology Research Center (protocol code 136 and 06/04/2018).

### 2.1 Study Population

The study included patients with non-valvular AF who were hospitalized in our clinic between 2019 and 2024 whose TEE results showed that they had LAAT for the first time before planned catheter ablation (CA) or elective cardioversion. The exclusion criteria were: (1) permanent AF, (2) a left ventricular (LV) ejection fraction of less than 50% according to transthoracic echocardiography (TTE), (3) unwillingness to participate in the study.

Patients with LAAT were then included in a follow-up study, the duration of which depended on the time of thrombus resolution (from 3 to 12 months) or was limited to 12 months. The end point of the observation was the resolution or persistence of LAAT. At the start of the study, all patients underwent a general clinical examination. Blood samples were drawn for CBC tests, biochemistry tests, and coagulogram parameters. Electrocardiography, TTE, and TEE were also performed.

### 2.2 Clinical Parameters

All participants underwent a complete physical evaluation. AF and comorbid diseases were diagnosed according to recommended guidelines. Paroxysmal AF was defined as self-terminating within 7 days of onset [1]. Persistent AF lasted longer than 7 days or required medication or electrical cardioversion for termination. Baseline data included sex, age, smoking and drinking habits, body mass index, hypertension, coronary artery disease, diabetes mellitus, heart failure (HF), and medication.

### 2.3 TTE and TEE

Both tests were conducted at the beginning of the study. All patients underwent TEE immediately prior to ablation or elective cardioversion to exclude LAAT. TTE was performed using a Vivid E9 ultrasound scanner (GE Medical Systems, Milwaukee, Wisconsin, USA) in accordance with the Recommendations of the American Society of Echocardiography and the European Association for Cardiovascular Imaging [11, 12], where chamber size and volume as well as systolic and diastolic LV function were assessed. TEE was performed by qualified cardiac sonographers using the Vivid E9 ultrasound scanner, a Vivid S70 ultrasound scanner (GE Medical Systems, Milwaukee, Wisconsin, USA), and a transesophageal matrix multiplane phased transducer. Left atrial appendix scanning was performed from the middle esophageal view between 0° and 110° in 10–20° increments.

The presence of thrombi in the LAA (and spontaneous echo contrast) was recorded and LAA flow velocity (LAAFV) was measured [13]. LAAFV was measured using a pulse-wave Doppler, with blood samples positioned 1 cm inside the LAA entrance. The average LAAFV value was determined from five consecutive cardiac cycles. LA thrombus was defined as a distinct echogenic mass separate from the LA body that could be acoustically differentiated from the surrounding atrium [4]. The presence of LA thrombus was confirmed by an independent observer who reviewed a recording of all studies. LAAT resolution was monitored by comparing the TEE data during follow-up with the initial one.

### 2.4 Blood Tests

Laboratory assessments included a CBC, biochemistry tests, lipid profiles, renal function, and coagulation. CBC parameters, namely WBC count and their subpopulations (i.e., neutrophils and lymphocytes); neutrophil-to-lymphocyte ratio; RBC count; hemoglobin; hematocrit (HCT); as well as PLT count and related factors, namely mean platelet volume (MPV), platelet distribution width (PDW), and platelet large cell ratio (P-LCR), were determined using a BC-6800 automated hematology analyzer (Mindray, Shenzhen, Guangdong, China).

Biochemistry tests were performed using a BS-480 clinical chemistry analyzer (Mindray, Shenzhen, Guangdong, China) and coagulation parameters were determined using a Destiny Plus automated coagulation analyzer (Tcoag Ireland Limited, Wicklow, Ireland).

N-terminal brain natriuretic propeptide (NT-proBNP) (reference value up to 125 pg/mL) was determined using chemiluminescent enzyme immunoassay on an IMMULITE 2000 analyzer (Siemens Diagnostics, Tarrytown, New York, USA).

### 2.5 Statistical Analysis

Statistical data analysis was performed using IBM SPSS Statistics (Version 21, IBM Corporation, Armonk, New York, USA) and Statistica (Version 12.0, StatSoft, Tulsa, Oklahoma, USA) programs. The distribution of quantitative variables was evaluated using the Shapiro-Wilk test or the Kolmogorov–Smirnov test. Under normal distribution, data is presented as the mean (M) plus the standard deviation (M ± SD). Otherwise, data is presented as the median (Me) and the interquartile range (IQR)–Me (Q1, Q3). A comparison of continuous variables in the groups was performed using the *T*-test in case of normal distribution and the Mann-Whitney U-test in case of other distribution.

Qualitative data were compared using Pearson’s $\chi^{2}$ test or Fisher’s exact test. Spearman’s correlation was used to analyze the relationship between variables. Univariate and multivariate logistic regression methods were used to find predictors of LAAT persistence and to obtain a model of the probability of its presence. Receiver operating characteristic (ROC) analysis was used to evaluate the quality and efficiency of the model. The results were evaluated as statistically significant at a two-way level of *p* < 0.05, and 0.05 < *p* < 0.1 was considered a statistical trend.

## 3. Results

**Fig. 1.**
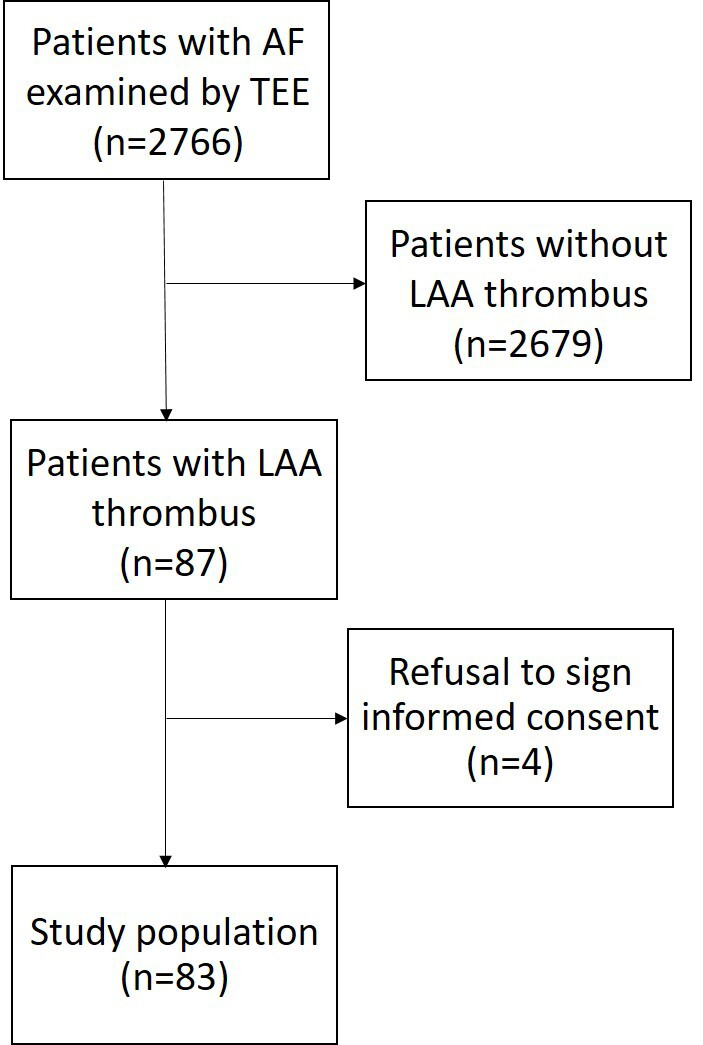
**Flow chart of the study protocol**. AF, atrial fibrillation; TEE, transesophageal echocardiography; LAA, left atrial appendage.

From 2019 to 2024, 2766 patients with non-valvular AF were hospitalized for elective CA or cardioversion. All patients underwent TEE to exclude LAAT. Of the 87 patients with LAAT, four were not included in the follow-up study due to their refusal to sign informed consent because of their unwillingness to repeat TEE; thus, 83 patients participated in the study. The patient selection process is illustrated in Fig. 1.

The median age of the patients was 62 (55, 65) years and 49 (59%) of the participants were men. Thirty-nine patients (47%) had paroxysmal AF while 44 patients (53%) had persistent AF. Patients were divided into two groups according to the results of repeat TEE: Group 1 (n = 45), in whom LAAT resolved within 12 months, and Group 2 (n = 38), in whom LAAT persisted until the end of the follow-up period. LAAT resolution occurred within the first three months of follow-up for 24 patients (53.3%) in Group 1, while LAAT resolution occurred after the three-month mark for the remaining 21 patients (46.7%). Baseline clinical characteristics and medications of the patients in the groups are presented in Table 1.

**Table 1.** **Baseline clinical characteristics and medications in the groups of patients**.

Characteristics		Group 1 (n = 45)	Group 2 (n = 38)	*p*-value
Age, years		61.1 ± 8.4	59.9 ± 7.3	0.490
Sex, male n (%)		26 (57.8)	23 (60.5)	0.800
Type of AF, n (%)				
	Paroxysmal	24 (53.3)	15 (39.5)	0.208
	Persistent	21 (46.7)	23 (60.5)	
BMI (kg/m^2^)		31.6 ± 5.7	32.7 ± 4.6	0.332
Hypertension, n (%)		43 (95.6)	36 (94.7)	1.000
Coronary artery disease, n (%)		30 (66.7)	23 (60.5)	0.562
History of MI, n (%)		1 (2.2)	1 (2.6)	1.000
Diabetes, n (%)		7 (15.6)	7 (18.4)	0.775
eGFR <60 mL/min/1.73 m^2^, n (%)		7 (15.6)	7 (18.4)	0.775
History of bleeding, n (%)		2 (4.4)	1 (2.6)	1.000
Medications				
	Amiodaron, n (%)	6 (13.3)	1 (2.6)	0.118
	Propafenon, n (%)	2 (4.4)	4 (10.5)	0.405
	Sotalol, n (%)	14 (31.1)	6 (15.8)	0.127
	Lappaconitine hydrobromide , n (%)	4 (8.9)	4 (10.5)	1.000
	B-blocker, n (%)	14 (31.1)	22 (57.9)	0.014*
	ARB and/or ACEi, n (%)	34 (75.6)	31 (81.6)	0.398
	Statins, n (%)	30 (66.7)	26 (70.3)	0.727
	Diuretics, n (%)	16 (35.6)	23 (60.5)	0.023*
	Calcium antagonists, n (%)	7 (15.6)	13 (35.1)	0.069^#^
Baseline OAC				
	Varfarin, n (%)	7 (15.5)	4 (10.5)	0.076
	Apixaban, n (%)	15 (33.3)	11 (28.9)	
	Rivaroxaban, n (%)	6 (13.3)	15 (39.5)	
	Dabigatran, n (%)	13 (28.9)	5 (13.2)	
Change of OAC				
	Total cases, n (%)	36 (80.0)	29 (76.3)	0.909
	DOAC to another DOAC, n (%)	29 (64.4)	25 (65.8)	
	DOAC to varfarin, n (%)	2 (4.5)	1 (2.6)	
	Varfarin to DOAC, n (%)	5 (11.1)	3 (7.9)	

**Abbreviations**: AF, atrial fibrillation; BMI, body mass index; MI, myocardial infarction; eGFR, estimated glomerular filtration rate; ARB, angiotensin receptor blocker; ACEi, angiotensinconverting enzyme inhibitor; OAC, oral anticoagulants; DOAC, direct OAC; *p-value < 0.05, ^#^*p*-value < 0.1.

### 3.1 Comparative Analysis of Clinical Data as Well as Echocardiographic and Hematological Parameters in Group 1 and Group 2 Patients 

There were no significant differences in age, gender, and incidence of cardiovascular diseases between patients in Groups 1 and 2. Patients who did not have LAAT lysis were more likely to take beta-blockers and diuretics, which is likely due to the need to choose a “heart rate control” strategy and the occurrence of HF. No statistically significant differences were found for the remaining medications. Of the anticoagulants, patients in the LAAT persistence group were more likely to be taking rivaroxaban at the start of the study. Sixty-five patients in whom thrombosis was detected for the first time while taking baseline oral anticoagulants (OAC) or with repeat TEE elected to change the type of oral anticoagulant when LAAT persistence was detected.

Comparative analysis of TTE and TEE parameters between the groups (Table 2) showed that patients with persistent LAAT had higher right atrial volume index (RAVi). Analysis also showed no significant differences in LA volume index. The LV posterior wall also tended to be thicker in patients with persistent LAAT.

**Table 2.** **Echocardiographic parameters of patients with lysed and persistent LAAT**.

Parameters	Group 1 (n = 45)	Group 2 (n = 38)	*p*-value
RAVi, mL/m^2^	24.5 (21.0, 32.2)	30.7 (24.7, 34.7)	0.034*
LAVi, mL/m^2^	36.6 (30.9, 46.4)	38.5 (31.3, 45.1)	0.942
LVESD, mm/m^2^	17.0 ± 3.0	17.0 ± 2.0	0.997
LVEDD, mm/m^2^	24.5 ± 2.8	24.3 ± 3.1	0.869
LVEDV, mL/m^2^	49.9 ± 11.0	52.8 ± 16.1	0.334
LVESV, mL/m^2^	18.1 (15.7, 24.6)	19.8 (15.4, 25.7)	0.759
IVS, mm	12.0 (11.0, 12.0)	12.0 (11.0, 13.0)	0.233
LVPW, mm	10.0 (10.0, 11.0)	11.0 (10.0, 12.0)	0.060^#^
LV mass index, g/m^2^	100.0 ± 17.6	107.4 ± 28.2	0.146
SV, mL	60.1 ± 13.7	65.4 ± 20.8	0.173
LVEF, %	60.0 (57.0, 64.0)	59.5 (55.0, 64.0)	0.787
PASP, mm Hg	27.0 (25.0, 30.0)	28.0 (25.0, 35.0)	0.730
LAAFV, сm/s	35.0 (30.0, 42.0)	32.0 (30.0, 36.0)	0.113

**Abbreviations**: RAVi, right atrial volume index; LAVi, left atrial volume index; LVESD, left ventricular end-systolic dimension; LVEDD, LV end-diastolic dimension; LVEDV, LV end-diastolic volume; LVESV, LV end-systolic volume; IVS, interventricular septum; LVPW, LV posterior wall; SV, stroke volume; LVEF, LV ejection fraction; PASP, pulmonary artery systolic pressure; LAAFV, LA appendage flow velocity; * - *p*-value < 0.05; # - *p*-value < 0.1.

Analysis of baseline hematological parameters (Table 3) showed statistically significant differences in the CBC parameters characterizing PLT morphofunctional indicators. MPV, PDW, and P-LCR were higher in patients with persistent LAAT compared to patients with resolved LAAT. The total WBC count, although within the reference range, was also higher in patients with persistent LAAT. The higher number of WBCs was due to both neutrophils and lymphocytes. Among other parameters, NT-proBNP tended to be higher in patients with persistent thrombus. Coagulation parameters did not differ between the groups.

**Table 3.** **Comparison of hematological parameters in patients with lysed and persistent LAAT**.

Parameters		Group 1 (n = 45)	Group 2 (n = 38)	*p*-value
CBC parameters				
	WBCs, 10^9^/L	5.7 ± 1.7	6.7 ± 1.8	0.014*
	RBCs, 10^12^/L	4.8 ± 0.5	5.0 ± 0.5	0.097^#^
	Hemoglobin, g/L	140.6 ± 15.7	146.5 ± 14.0	0.083^#^
	HCT, %	43.8 ± 5.3	45.3 ± 4.4	0.160
	PLTs, 10^9^/L	211.0 ± 45.4	224.3 ± 64.3	0.276
	Plateletcrit, %	0.17 (0.15, 0.19)	0.20 (0.15, 0.22)	0.266
	MPV, fL	8.4 (7.9, 9.4)	9.1 (8.3, 9.8)	0.035*
	PDW, %	15.7 (15.5, 15.9)	15.9 (15.7, 16.2)	0.007*
	P-LCR, %	25.3 ± 7.4	30.0 ± 9.2	0.014*
	Neutrophils, 10^9^/L	3.0 (2.4, 4.0)	3.6 (3.0, 4.3)	0.018*
	Lymphocytes, 10^9^/L	1.7 (1.4, 2.2)	2.1 (1.6, 2.6)	0.018*
	NLR	1.8 (1.2, 2.2)	1.7 (1.4, 2.0)	0.981
Biochemistry tests				
	FBG, mmol/L	5.7 (5.2, 6.2)	5.6 (5.3, 6.4)	0.955
	Creatinine, µmol/L	86.0 (79.0, 91.0)	85.4 (77.0, 101.0)	0.531
	eGFR, mL/min/1.73 m^2^	75.8 ± 15.2	73.7 ± 12.7	0.507
	АST, U/L	23.4 (19.8, 27.5)	22.2 (17.6, 27.4)	0.635
	ALT, U/L	24.5 (20.2, 38.5)	26.1 (19.4, 37.1)	0.680
	Total cholesterol, mmol/L	4.3 ± 1.0	4.5 ± 1.0	0.294
	HDL-C, mmol/L	1.3 (1.1, 1.5)	1.2 (1.0, 1.4)	0.512
	LDL-C, mmol/L	2.4 (1.9, 2.9)	2.6 (2.1, 3.1)	0.162
	Triglyceride, mmol/L	1.2 (1.0, 1.5)	1.3 (1.1, 1.8)	0.457
	CRP, mg/L	1.9 (1.1, 4.4)	1.9 (0.9, 3.7)	0.563
	NT-proBNP, pg/mL	280.0 (77.8, 639.0)	599.0 (128.0, 1656.0)	0.058^#^
Coagulation parameters				
	APTT, sec	33.7 (30.8, 38.7)	34.5 (30.8, 40.5)	0.625
	Fibrinogen, g/L	3.1 ± 0.5	3.0 ± 0.6	0.652
	Thrombin clotting time	18.6 (17.0, 26.6)	18.6 (17.1, 26.0)	0.731
	D-dimer, ng/L	0.39 (0.18, 0.37)	0.29 (0.23, 0.40)	0.374
	Antithrombin III, %	93.2 ± 20.8	93.4 ± 21.4	0.974
	Prothrombin index, %	82.9 (72.6, 91.6)	78.6 (67.5, 87.0)	0.235

**Abbreviations**: CBC, complete blood count; WBCs, white blood cells; RBCs, red blood cells; HCT, hematocrit; PLTs, platelets; MPV, mean platelet volume; PDW, platelet distribution width; P-LCR, platelet large cell ratio; NLR, Neutrophil-to-Lymphocyte Ratio; FBG, fasting blood glucose; eGFR, estimated glomerular filtration rate; AST, aspartate aminotransferase; ALT, alanine aminotransferase; HDL-C, high-density lipoprotein cholesterol; LDL-C, low-density lipoprotein cholesterol; CRP, C-reactive protein; NT-proBNP, N-terminal pro-brain natriuretic peptide; APTT, activated partial thromboplastin time. * - *p*-value < 0.05; # - *p*-value < 0.1.

### 3.2 Analysis of Correlations Between Platelet Parameters and Other Indicators

The results of the correlation analysis are presented in Table 4.

**Table 4.** **Correlation analysis results**.

Parameters	MPV		PDW		P-LCR	
	rs	*p*-value	rs	*p*-value	rs	*p*-value
WBCs, 10^9^/L	0.219	0.048	–0.042	NS	0.160	NS
RBCs, 10^12^/L	0.277	0.012	0.130	NS	0.248	0.029
Hemoglobin, g/L	0.267	0.015	0.176	NS	0.226	0.049
HCT, %	0.294	0.007	0.234	0.039	0.302	0.008
PLTs, 10^9^/L	–0.239	0.030	–0.453	0.000	–0.376	0.001
Plateletcrit, %	0.130	NS	–0.253	0.026	0.042	NS
Lymphocytes, 10^9^/L	0.225	0.044	–0.061	NS	0.188	0.089
RAVi, mL/m^2^	0.290	0.009	0.203	0.081	0.308	0.008
PASP, mm Hg	0.234	0.036	0.128	NS	0.267	0.021
NT-proBNP, pg/mL	0.162	NS	0.237	0.048	0.259	0.032

**Abbreviations**: MPV, mean platelet volume; PDW, platelet distribution width; P-LCR, platelet large cell ratio; rs, Spearman correlation coefficient; WBCs, white blood cells; RBCs, red blood cells; HCT, hematocrit; PLTs, platelets; RAVi, right atrial volume index; PASP, pulmonary artery systolic pressure; NT-proBNP, N-terminal pro-brain natriuretic peptide; NS, not statistically significant.

Platelet parameters had direct correlations with WBC count, RBC count, hemoglobin, and HCT. Platelet parameters also positively correlated with indicators characterizing the severity of HF, such as pulmonary artery systolic pressure and NT-proBNP. All three parameters were directly correlated with RAVi, which may indicate the severity of atrial structural remodeling associated with HF with preserved LV ejection fraction (HFpEF).

### 3.3 Search for Predictors of LAAT Resistance to Lysis

To create a model capable of predicting LAAT persistence over 12 months, several variables that showed significant or close to significant differences when the groups were compared were included in univariate and then in multivariate logistic regression analysis.

To obtain the final model with optimal characteristics, multivariate logistic regression analysis included combinations of variables that were significantly related to LAAT persistence according to univariate logistic regression but not related to each other (i.e., MPV, PDW, P-LCR, as well as neutrophils, lymphocytes, and WBCs) were not simultaneously included in the analysis.

As a result, a predictive model with an optimal sensitivity and specificity ratio was obtained. The results are presented in Table 5.

**Table 5.** **Results of logistic regression analysis**.

Variables	Univariate analysis			Multivariate analysis		
	OR	95% CI	*p*-value	OR	95% CI	*p*-value
B-blockers intake	3.045	1.236–7.501	0.016	-	-	-
Diuretics intake	2.779	1.139–6.781	0.025	3.800	1.281–11.275	0.016
RBCs	2.052	0.873–4.823	0.099	-	-	-
WBCs, 10^9^/L	1.380	1.056–1.804	0.018	-	-	-
Neutrophils	1.443	0.961–2.167	0.077	-	-	-
Lymphocytes	2.039	0.990–4.201	0.053	-	-	-
Hemoglobin	1.027	0.996–1.059	0.086	-	-	-
MPV, fL	1.764	1.096–2.837	0.019	1.892	1.056–3.387	0.032
PDW	3.351	0.944–11.895	0.061	-	-	-
P-LCR	1.077	1.012–1.146	0.020	-	-	-
RAVi, mL/m^2^	1.029	0.979–1.081	0.262	-	-	-
LVPW	1.494	1.005–2.221	0.049	-	-	-
NT-proBNP, pg/mL	1.001	1.000–1.001	0.018	1.001	1.000–1.001	0.015

**Abbreviations**: OR, odds ratio; CI, confidence interval; RBCs, red blood cells; WBCs, white blood cells; MPV, mean platelet volume; PDW, platelet distribution width; P-LCR, platelet large cell ratio; RAVi, right atrial volume index; LVPW, LV posterior wall; NT-proBNP, N-terminal pro-brain natriuretic peptide.

**Fig. 2.**
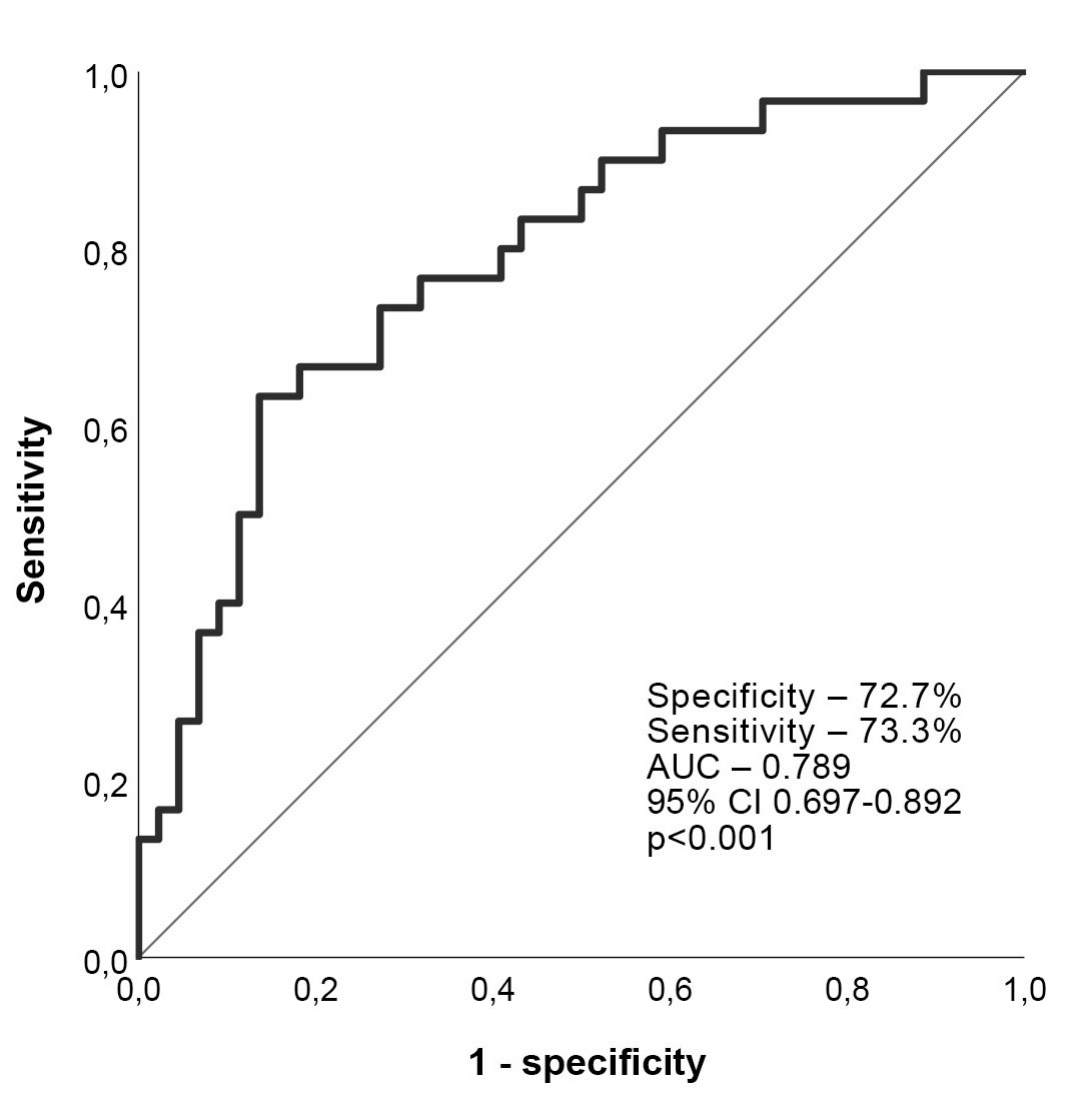
**ROC curve analysis**. AUC, Area under the ROC curve.

Multivariate logistic regression analysis showed that diuretic intake (*p* = 0.016), MPV (*p *= 0.032), and NT-proBNP level (*p* = 0.015) are independent predictors of LAAT resistance to resolution, regardless of the type of baseline OAC or change in OAC. ROC analysis confirmed the good quality of the resulting model (Fig. 2): AUC—0.789, specificity—72.7%, sensitivity—73.3%, predictive accuracy—73.0%.

Statistical analysis showed that a 100 pg/mL increase in NT-proBNP level increased the risk of LAAT resistance to lysis by 8.3%, and a 1 unit (1 fL) increase in MPV was associated with an 89% increase in the risk of LAAT resistance.

## 4. Discussion 

The problem of effective LAAT lysis in patients with non-valvular AF raises many questions since current guidelines do not have clear recommendations on the methods and timing of treatment of such patients. Identifying factors that prevent LAAT resolution is important, as it is an absolute contraindication to CA. There are limited studies on thrombolytic treatment for LAAT resolution, and there have been no large randomized clinical trials to guide treatment [14].

It has been reported that the incidence of LAAT in patients with AF ranges from 0.5% to 14.0% [15]. In our study, the incidence of LAAT and the proportion of persistent LAAT are generally consistent with other studies. According to our data, LAAT was detected in 3.1% of patients referred for CA or electrical cardioversion and persisted during the 12-month follow-up in 45.8% of them. This is comparable to the results obtained by Bernhardt *et al*. (2004) [16], according to which thrombolysis was also absent in 44.0% of patients after 1 year of follow-up, despite antithrombotic therapy.

All of our patients took OAC before primary TEE, with the majority taking DOAC. There were no differences between groups in the type of OAC, except that a larger proportion of Group 2 patients took rivaroxaban. There were also no differences between the groups in the proportion of patients who changed OAC during the follow-up study. We considered it incorrect to include the type of OAC in the list of predictors since this was not originally stipulated in the protocol and no randomization of patients by type of OAC was conducted.

A literature search found several publications devoted to the study of factors contributing to LAAT persistence; however, these studies were primarily devoted to evaluating the efficacy of various OACs in resolving LAAT. In one such meta-analysis, Cheng *et al*. (2022) [7] showed that none of the inter-study variables for VKA and DOAC significantly predicted the frequency of LAAT resolution. In another meta-analysis, Mo *et al*. (2025) [14] studied AF subtype, LA diameter, and LV diastolic diameter as potential factors influencing LA thrombus resolution. They found that although effective thrombolysis was associated with smaller LA diameter (*p* = 0.04) and larger LV diastolic diameter (*p* < 0.00001), it was independent of AF subtype (*p* = 0.16) and LV ejection fraction (*p* = 0.720) [14].

In our study, diuretic intake, NT-proBNP, and MPV were identified as potential predictors of LAAT resistance to thrombolysis in patients with AF regardless of type of OAC. The first two predictors can be explained by the more pronounced HFpEF in Group 2 patients, which was confirmed by a higher RAVi as well as a tendency towards higher NT-proBNP levels. A main finding in our study was that MPV, a routine CBC parameter, appeared to be an independent predictor of LAAT resistance to resolution.

Studies have shown that the prothrombotic status in patients with AF is a pathological condition caused by various factors, such as disorders of the hemostasis system, coagulation and the anticoagulant system [17]. Platelets activation is one of the components of prothrombotic status. Peripheral PLT consumption contributes to an increase in the number of newly formed immature PLTs, which are larger and more reactive than their mature counterparts [18]. It has also been hypothesized that there is a correlation between PLT size and content. It is assumed that larger PLTs contain more granules, the most numerous of which are alpha granules, making them more reactive. Fibrinogen, von Willebrand factor, thrombospondin, thromboxane A2, and transforming growth factor are largely preserved in these granules [19].

MPV is an accurate marker of PLT size and is more convenient and economical to measure than other PLT activation markers [20]. MPV is calculated by dividing the plateletcrit by the total number of PLTs; therefore, higher MPV indicates higher PLT turnover [19]. Studies have shown that high MPV levels is independently associated with thrombosis [21, 22]. MPV has also been shown to be a predictor of ischemic stroke in patients with AF [9, 23]. In our study, MPV level did not exceed the reference level in either group; however, it was significantly higher in the group with resistant thrombus. This may indicate a persistent prothrombotic status in these patients despite OAC administration.

It should also be noted that MPV has been proven to be a predictor of HFpEF severity, and elevated MPV levels have been associated with poor outcomes in patients with HFpEF [19]. In our study, there was a correlation between MPV and indicators such as pulmonary artery systolic pressure and RAVi, which suggests that HFpEF severity may be a primary indicator of LAAT resistance to lysis. HFpEF is known to be associated with a proinflammatory status caused by comorbidities, with an impact on endothelium function, involving complex molecular pathways that ultimately lead to myocardial fibrosis and LV dysfunction [24]. Inflammatory processes promote the release of cytokines, reorganization of the extracellular matrix, and PTL activation, supporting the potential role of PTLs in the pathophysiology of HFpEF [25]. Therefore, in such patients, primary efforts should be directed towards the treatment of HFpEF.

The results obtained confirm the need for further research aimed at studying the factors contributing to the resistance of LAAT to lysis, as well as finding ways to overcome this resistance.

### Limitations

Our study has several limitations. First, this study was conducted at a single medical center in the Russian Federation. Despite a fairly large number of patients during screening, the sample in the study was small for an objective reason related to the low incidence of LAA thrombosis in patients with AF while taking anticoagulants. The small sample size also prevented us from analyzing the effect of type of oral anticoagulants and change of oral anticoagulants on the effectiveness of thrombosis resolution. It should also be noted that only patients with preserved LV ejection fraction (>50%) were included in our study. Since the study design was observational, the associations we identified between MPV and LAAT resistance, do not allow us to establish causality between them.

## 5. Conclusion

This study confirmed that in patients with AF, approximately 47% of LAAT cases remains resistant to lysis 1 year after initial detection. In this work, it was revealed for the first time that higher PLTs parameters, in particular MPV, are associated with LAAT resistance, and continued research in this direction is required.

## Data Availability

The datasets used and analyzed during the current study are available from the corresponding author on reasonable request.

## Abbreviations

AF, atrial fibrillation; CA, catheter ablation; CBC, complete blood count; DOAC, direct oral anticoagulants; HF, heart failure; HFpEF, HF with preserved LV ejection fraction; HCT, hematocrit; RAVi, right atrial volume index; LAA, left atrial appendage; LAAFV, LAA flow velocity; LAAT, left atrial appendage thrombosis; LV, left ventricular; MPV, mean platelet volume; NT-proBNP, N-terminal brain natriuretic propeptide; OAC, oral anticoagulants; PDW, platelet distribution width; P-LCR, platelet large cell ratio; PLTs, platelets; RBCs, red blood cells; TEE, transoesophageal echocardiography; TTE, transthoracic echocardiography; VKA, vitamin K antagonists; WBCs, white blood cells.
